# Relationship Between Reactive Astrocytes, by [^18^F]SMBT-1 Imaging, with Amyloid-Beta, Tau, Glucose Metabolism, and TSPO in Mouse Models of Alzheimer’s Disease

**DOI:** 10.1007/s12035-024-04106-7

**Published:** 2024-03-19

**Authors:** Yanyan Kong, Cinzia A. Maschio, Xuefeng Shi, Fang Xie, Chuantao Zuo, Uwe Konietzko, Kuangyu Shi, Axel Rominger, Jianfei Xiao, Qi Huang, Roger M. Nitsch, Yihui Guan, Ruiqing Ni

**Affiliations:** 1 PET Center, Huashan Hospital, Fudan University, Shanghai, China; 2 Institute for Regenerative Medicine, University of Zurich, Zurich, Switzerland; 3 Qinghai Provincial People’s Hospital, Xining, China; 4 Zurich Neuroscience Zentrum (ZNZ), Zurich, Switzerland; 5 Department of Nuclear Medicine, Inselspital, University of Bern, Bern, Switzerland; 6 Institute for Biomedical Engineering, University of Zurich & ETH Zurich, Zurich, Switzerland

**Keywords:** Alzheimer’s disease, Amyloid-beta, Glia, MAO-B, PET, Tau, TSPO

## Abstract

Reactive astrocytes play an important role in the development of Alzheimer’s disease (AD). Here, we aimed to investigate the temporospatial relationships among monoamine oxidase-B, tau and amyloid-β (Aβ), translocator protein, and glucose metabolism by using multitracer imaging in AD transgenic mouse models. Positron emission tomography (PET) imaging with [^18^F]SMBT-1 (monoamine oxidase-B), [^18^F]florbetapir (Aβ), [^18^F]PM-PBB3 (tau), [^18^F]fluorodeoxyglucose (FDG), and [^18^F]DPA-714 (translocator protein) was carried out in 5- and 10-month-old APP/PS1, 11-month-old 3×Tg mice, and aged-matched wild-type mice. The brain regional referenced standard uptake value (SUVR) was computed with the cerebellum as the reference region. Immunofluorescence staining was performed on mouse brain tissue slices. [^18^F]SMBT-1 and [^18^F]florbetapir SUVRs were greater in the cortex and hippocampus of 10-month-old APP/PS1 mice than in those of 5-month-old APP/PS1 mice and wild-type mice. No significant difference in the regional [^18^F]FDG or [^18^F]DPA-714 SUVRs was observed in the brains of 5- or 10-month-old APP/PS1 mice or wild-type mice. No significant difference in the SUVRs of any tracer was observed between 11-month-old 3×Tg mice and age-matched wild-type mice. A positive correlation between the SUVRs of [^18^F]florbetapir and [^18^F]DPA-714 in the cortex and hippocampus was observed among the transgenic mice. Immunostaining validated the distribution of MAO-B and limited Aβ and tau pathology in 11-month-old 3×Tg mice; and Aβ deposits in brain tissue from 10-month-old APP/PS1 mice. In summary, these findings provide in vivo evidence that an increase in astrocyte [^18^F]SMBT-1 accompanies Aβ accumulation in APP/PS1 models of AD amyloidosis.

**Supplementary Information:**

The online version contains supplementary material available at 10.1007/s12035-024-04106-7.

## Introduction

Alzheimer’s disease (AD) is pathologically characterized by abnormal accumulation of amyloid-beta (Ab), tau tangles, reactive astrocytes, microgliosis, and neuronal loss. Astrocytes are the most abundant glial cell population in the brain and play an important role in maintaining synaptic homeostasis by regulating synapse function, calcium signalling, and brain metabolism [80]. Reactive astrocytes are involved early in the pathophysiology of AD and have a dynamic profile during disease progression [6, 23]. Postmortem studies of AD brains have demonstrated abundant reactive astrocytes and microglia around Aβ plaques and tangles [51, 57, 71]. Previous topological analyses revealed that astrocytes respond to plaque-induced neurological injury primarily by changing their phenotype and hence function rather than their location [28]. The heterogeneity of astrocyte and microglial profiles in these models has been documented in earlier transcriptomic studies. Reactive astrocytes with altered metabolism and function have been demonstrated in an amyloidosis animal model [2]. Reactive astrocytes acquire neuroprotective and deleterious signatures in response to tau and Aβ pathology [36] and influence the effects of amyloid-β on tau pathology in preclinical AD [5]. Reactive astrocytes, as measured by cerebrospinal fluid (CSF) levels of glial fibrillary acidic protein (GFAP), have been shown to mediate the effect of Aβ on tau and drive downstream neurodegeneration and cognitive impairment in patients with AD [24] and preclinical AD. Monoamine oxidase B (MAO-B) is expressed mainly on astrocytes but also on serotoninergic and histaminergic neurons. MAO-B reversibly increases astrocytic γ-aminobutyric acid (GABA) production in reactive astrocytes [37], which is associated with synaptic and memory impairments in APP/PS1 mice with amyloidosis [63]. Moreover, MAO-B mediates the aberrant synthesis of hydrogen peroxide (H_2_O_2_) in reactive astrocytes. There is an age-related increase in MAO-B expression in astrocytes [77]. Furthermore, the levels of MAO-B have been shown to increase in the brains of sporadic and autosomal dominant AD patients and mild cognitive impairment patients. A recent study showed that MAO-B is elevated in AD pyramidal neurons, is associated with γ-secretase, and regulates neuronal Aβ-peptide levels [70]. In APP/PS1 mice, upregulated levels of MAO-B and reactive astrocytes increase the number of tau inclusions, increase neuronal death and brain atrophy, and impair spatial memory in an H_2_O_2_-dependent manner [17]. Reactive astrocytes and MAO-B have thus emerged as potential treatment targets for AD [72].

Several positron emission tomography (PET) tracers for reactive astrocytes have been developed, including the irreversible MAO-B tracers [^11^C]deuterium-L-deprenyl (DED) and [^18^F]F-DED [3, 60, 66], the reversible MAO-B tracer [^18^F]SMBT-1, the substrate-based MAO-B tracer [^11^C]Cou [21], the mitochondrial imidazoline 2 binding site (I_2_BS) tracer [^11^C]BU99008 [24] and [^11^C]acetate [53], and the thyroid hormone transporter OATP1C1 [^18^F]sulforhodamine-101 [45]. In vivo [^11^C]DED has demonstrated divergent longitudinal changes in reactive astrocytes and amyloid in patients with autosomal dominant [81] and prodromal AD [10, 67]. Increased brain [^18^F]SMBT-1 binding in Aβ + patients compared with that in Aβ-nondemented controls has been observed and is associated with Aβ accumulation at the preclinical stage of AD [13, 31, 82, 83]. Moderate correlations were found between [^11^C]DED and [^11^C]PIB and [^18^F]FDG [10, 69]. In animal models (APPswe, PS2APP, APPArcSwe), reactive astrocytes measured by using [^11^C]DED and [^18^F]F-DED precede the increase in the amyloid-PET signal [3, 60, 66]. Regional dependency of [^18^F]SMBT-1 and [^11^C]DED binding has been reported in the human brain by PET in vivo and by autoradiography on postmortem brain tissue, with the highest uptake in the striatum and thalamus, followed by the hippocampus and cortical regions; white matter; and rather low uptake in the cerebellum [26, 29, 31]. Moreover, MAO-B is also particularly enriched in the superficial layer [77].

The aim of the current study was to evaluate the distribution of the novel tracer [^18^F]SMBT-1 in two mouse models of AD (APP/PS1, 3×Tg). We assessed the temporospatial relationship of astrocyte MAO-B with alterations in Aβ accumulation (by [^18^F]florbetapir), tau levels (by [^18^F]PM-PBB3, florzolotau, APN-1607), glucose metabolism (by [^18^F]fluorodeoxyglucose, FDG), and translocator protein (TSPO, by [^18^F]DPA-714) using a multitracer approach. We hypothesized that MAO-B increase (reactive astrocytes) is associated with Aβ accumulation in mouse model of amyloidosis.

## Methods

### Animal Models

The animal models used in the study are summarized in Table 1. 3×Tg mice [B6;129-Psen1tm1MpmTg(APPSwe, tauP301L)1Lfa/Mmjax] aged 11 months [59], and APP/PS1 mice [B6. Cg-Tg(APPswe,PSEN1dE9)85Dbo/Mmjax] mice overexpressing the human APP695 transgene (Swedish (K670N/M671L)) and with PSEN1 mutations [34] aged 5 and 10 months were used (Jax Laboratory, USA). Wild-type C57BL6 mice were obtained from Charles River, Germany, and Cavins Laboratory Animal Co., Ltd., of Changzhou. Mice were housed in ventilated cages inside a temperature-controlled room under a 12-h dark/light cycle. Pelleted food (3437PXL15, CARGILL) and water were provided ad libitum. Paper tissue and red Tecniplast Mouse House® (Tecniplast, Italy) shelters were placed in cages for environmental enrichment.

**Table 1:** Information on the animal models used in the study

Mice	Age (month)	[^18^F]SMBT-1	[^18^F]DPA-714	[^18^F]PM-PBB3	[^18^F]florbetapir	[^18^F]FDG	[^11^C]PIB
APP/PS1	5	6 M	6 M	3 M	10 M	6 M	
	10	6 M	6 M	3 M	9 M	6 M	
3×Tg	11	8 M	3 M	5 M	3 M	3 M	2F/2 M
Wildtype	5	9 M	9 M	9 M	8 M	6 M	
	10	8 M	6 M	6 M	10 M	6 M	4 M

*F*, female; *M*, male

### Radiosynthesis

[^18^F]SMBT-1 (0.74 GBq/ml) was radiosynthesized from its precursor according to previous methods [31]. [^18^F]DPA-714 (1.48 GBq/ml) was labelled with ^18^F at its 2-fluoroethyl moiety after nucleophilic substitution of the corresponding linear analog [32]. [^18^F]PM-PBB3 (1.48 GBq/ml) was synthesized from an automatic synthesis module and kit provided by APRINOIA therapeutics (Suzhou, China) [39, 44]. [^18^F]florbetapir (0.56 GBq/ml) was radiosynthesized from its precursor in a fully automated procedure suitable for routine clinical application [49]. [^18^F]FDG (1.48 GBq/ml) was prepared in the radiochemistry facility of the PET Center, Huashan Hospital, Fudan University, for clinical use under Good Manufacturing Practices requirements. [^11^C]PIB (0.074 GBq/ml) was radiosynthesized according to a previously described protocol [86]. The identities of the aforementioned final products were confirmed by comparison with the high-performance liquid chromatography (HPLC) retention times of the nonradioactive reference compounds obtained by coinjection using a Luna 5 μm C18(2) 100 Å (250 mm × 4.6 mm) column (Phenomenex) with acetonitrile and water (60:40) as the solvent at a 1.0 mL/min flow rate. A radiochemical purity > 95% was achieved for all the aforementioned tracers. The HPLC and quality control (QC) data of [^18^F]SMBT-1 are shown in SFig. 1.

### MicroPET

PET experiments using [^18^F]SMBT-1, [^18^F]florbetapir, [^18^F]PM-PBB3, [^18^F]FDG, and [^18^F]DPA-714 were sequentially performed using a Siemens Inveon PET/CT system (Siemens Medical Solutions, United States) [43]. There was two days of rest between each scan. To confirm the results of [^18^F]florbetapir imaging in 3×Tg mice, [^11^C]PIB was also performed on four 3×Tg mice and four wild-type mice. Prior to the scans, the mice were anesthetized using isoflurane (1.5%) in medical oxygen (0.3–0.5 L/min) at room temperature with an isoflurane vaporizer (Molecular Imaging Products Company, USA). The mice were positioned in a spreadup position on the heated imaging bed and subjected to inhalation of the anesthetic during the PET/computed tomography (CT) procedure. The temperature of the mice was monitored. A single dose of tracer (∼0.37 MBq/g body weight, 0.1–0.2 mL) was injected into the animals through the tail vein under isoflurane anesthesia. For dynamic PET, the raw PET data were binned into nine frames (9 × 600 s) to obtain the time activity curve ([^18^F]SMBT-1 and [^18^F]PM-PBB3). Static PET/CT images were obtained for a 10-min period at specific times post intravenous administration, depending on the tracer used: [^18^F]FDG at 60–70 min, [^18^F]florbetapir at 50–60 min, [^18^F]SMBT-1 at 60–70 min, [^18^F]DPA-714 at 40–50 min, [^18^F]PM-PBB3 at 90–100 min, and [^11^C]PIB at 50–60 min. PET/CT images were reconstructed using the ordered subsets expectation maximization 3D algorithm (OSEM3D), with a matrix size of 128 × 128 × 159 and a voxel size of 0.815 mm × 0.815 mm × 0.796 mm. The data were reviewed using Inveon Research Workplace software (Siemens). Attenuation corrections derived from hybrid CT data were applied.

### PET Data Analysis

The images were processed and analyzed using PMOD 4.4 software (PMOD Technologies Ltd., Switzerland) by two people. Radioactivity is presented as the standardized uptake value (SUV) (decay-corrected radioactivity per cm^3^ divided by the injected dose per gram body weight). The time − activity curves were deduced from specific volumes of interest that were defined based on a mouse MRI T_2_-weighted image template [81]. The brain regional SUVRs were calculated using the cerebellum (Cb) as the reference region. The mask was applied for signals outside the brain volumes of interest for illustration.

### Immunofluorescence Staining

Mice were perfused under ketamine/xylazine/acepromazine maleate anesthesia (75/10/2 mg/kg body weight, i.p. bolus injection) with ice-cold 0.1 M phosphate-buffered saline (PBS, pH 7.4) and 4% paraformaldehyde (PFA) in 0.1 M PBS (pH 7.4), fixed for 24 h in 4% PFA and then stored in 0.1 M PBS at 4 °C. The APPS/PS1 mice used for staining were subjected to in vivo imaging. The 3×Tg mice were purchased from the same source (Jax Laboratory) but different for in vivo and ex vivo experiments. Sagittal and coronal brain Sects. (40 mm) were cut around bregma 0 to -2 mm. The sections were first washed in PBS 3 × 10 min, followed by antigen retrieval for 20 min in citrate buffer at room temperature. Then, the sections were permeabilized and blocked in 5% normal donkey or goat serum and 1% Triton-PBS for one hour at room temperature. Free-floating tissue sections were incubated with primary antibodies against 6E10, complement component C3d (C3D), CD68, AT-8, GFAP, glucose transporter type-1 (Glut1), MAO-B overnight at 4 °C (Suppl. Table 1) [40] and with the appropriate secondary antibodies. The sections were incubated for 15 min in 4’,6-diamidino-2-phenylindole (DAPI), washed 2 × 10 min with PBS, and mounted with VECTASHIELD Vibrance Antifade Mounting Media (Vector Laboratories, Z J0215). The brain sections were imaged at × 20 magnification using an Axio Oberver Z1 slide scanner (Zeiss, Germany) using the same acquisition settings for all slices and at × 10 and × 63 magnification using a Leica SP8 confocal microscope (Leica, Germany). The images were analyzed by a person blinded to the genotype using Qupath and ImageJ (NIH, U.S.A.).

### Statistics

Two-way ANOVA with Sidak post hoc analysis was used for comparisons between groups (GraphPad Prism 9.0, CA, USA). Nonparametric Spearman’s rank correlation analysis was used to evaluate the associations between the regional SUVRs of different tracers. *P* < 0.05 indicated statistical significance. The data are presented as the mean ± standard deviation.

## Results

### *Higher Regional [*^*18*^*F]Florbetapir SUVRs in the Brains of APP/PS1 Mice at 5 and 10 Months of Age*

**Fig. 1:**
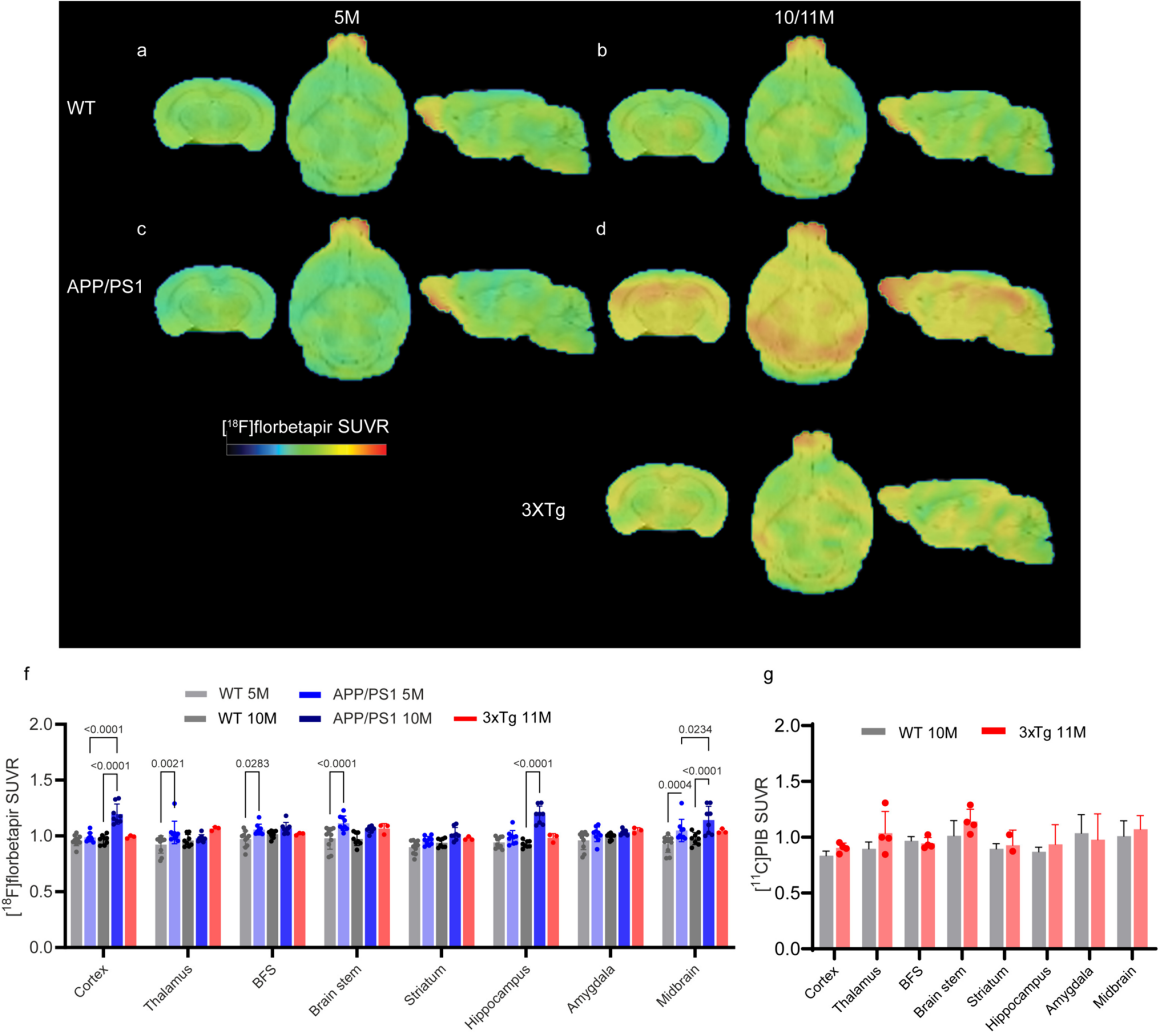
Increased [^18^F]florbetapir brain uptake in 10-month-old APP/PS1 mice compared to 5-month-old APP/PS1 mice and age-matched wild-type mice and 3×Tg mice. **a-e** Images of SUVRs from 5- and 10-month-old wild-type (WT, **a**, **b**), 5- and 10-month-old APP/PS1 (**c**, **d**), and 11-month-old 3×Tg mice (**e**). The SUVR scale was 0–2.2. **f** Quantification of [^18^F]florbetapir in WT, APP/PS1, and 3×Tg mice using Cb as the reference region. **g** There was no difference in [^11^C]PIB brain uptake between 3×Tg mice and wild-type mice. The SUVR was calculated using the Cb as the reference brain region. BFS, basal forebrain system; BFS, basal forebrain system; Cb, cerebellum

APP/PS1 mice and 3×Tg mice both develop plaque at approximately 6 months of age [34, 59]; however, recent characterization of 3×Tg mice revealed very little pathology at 12 months [35] Therefore, we chose to investigate the APP/PS1 mice at 5 and 10 months to represent the preplaque and plaque, respectively, and 3×Tg mice at 11 months. We first assessed the distribution of amyloid pathology in these mice using [^18^F]florbetapir PET. We used the cerebellum as a reference region for the quantification of the SUVR, as in earlier studies using [^18^F]florbetapir imaging in APP/PS1 model [27]. The [^18^F]florbetapir SUVR (Cb as the reference region) was greater in the thalamus, basal forebrain system, brainstem, and midbrain of 5-month-old APP/PS1 mice than in age-matched wild-type mice. A greater [^18^F]florbetapir SUVR was observed in the cortex and hippocampus of 10-month-old APP/PS1 mice than in age-matched wild-type mice and 5-month-old APP/PS1 mice (Fig. 1). In contrast, no regional differences in [^18^F]florbetapir SUVR were observed between the brains of 11-month-old 3×Tg mice and age-matched wild-type mice (Fig. 1e).

To further support our negative [^18^F]florbetapir results in the brain of 3×Tg mice, we performed PET using another amyloid tracer, [^11^C]PIB, in four 3×Tg mice. We used the cerebellum as the reference region for the quantification of [^11^C]PIB SUVRs in the mouse brain [73]. No difference in the [^11^C]PIB SUVR was observed in the brains of 11-month-old 3×Tg mice compared to age-matched wild-type mice, which is in line with our observation by using [^18^F]florbetapir (Fig. 1f). No significant between-group differences was observed in [^18^F] florbetapir SUVR in the olfactory bulb (SFig. 4a). No significant between-group differences was observed in [^18^F]florbetapir SUV in the cerebellum (SFig. 4f).

### *No Difference in [*^*18*^*F]PM-PBB3 SUVRs in the Cortex or Hippocampus of APP/PS1 Mice and 3*×*Tg Mice Compared to WT Mice*

**Fig. 2:**
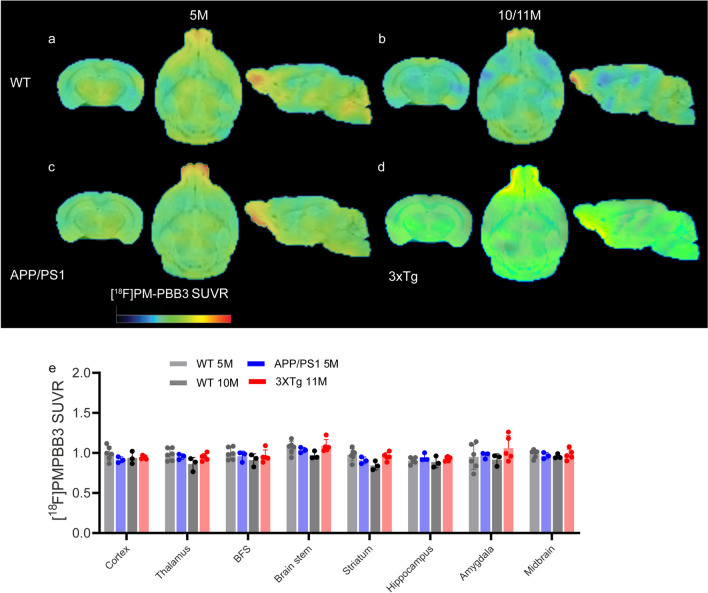
[^18^F]PM-PBB3 brain uptake did not differ between 3×Tg mice and age-matched wild-type mice. **a-d** Images of SUVRs from 5- and 10-month-old wild-type (WT, **a**, **b**), 5-month-old APP/PS1 (**c**), and 11-month-old 3×Tg mice (**d**). The SUVR scale was 0–2.2. **e** Quantification of [^18^F]PM-PBB3 in WT and 3×Tg mice using Cb as the reference brain region. BFS, basal forebrain system; Cb, cerebellum

The initial study on 3×Tg mice showed that tau deposits developed at 9 months [59]; however, recent characterization suggested that there is a lack of AT-8-positive signals in the hippocampus [35]. In vivo Tau PET has not been reported in 3×Tg mice. We therefore characterized the tau distribution in the brains of APP/PS1 mice at 5 months (as another control group) and 3×Tg mice at 11 months by PET using [^18^F]PM-PBB3. [^18^F]PM-PBB3 has been used to study tau distribution in tau mouse models [76]. The cerebellum was validated in earlier studies as a reference region for the quantification of the [^18^F]PM-PBB3 SUVR [76, 85]. We chose 90 min postinjection for the [^18^F]PM-PBB3 static scan based on the time activity curve (SFig. 2) and previous studies [76, 85]. The Cb was used as the reference brain region, as in previous PET studies with [^18^F]PM-PBB3 [76]. No regional difference in [^18^F]PM-PBB3 SUVR was observed in the brains of 5-month-old APPPS1 mice or 11-month-old 3×Tg mice compared to age-matched wild-type mice (Fig. 2). No significant between-group differences was observed in [^18^F]PM-PBB3 SUVR in the olfactory bulb (SFig. 4b). No significant between-group differences was observed in [^18^F]PM-PBB3 SUV in the cerebellum (SFig. 4 g).

### *Increased [*^*18*^*F]SMBT-1 SUVRs in the Cortex and Hippocampus of 10-month-old APP/PS1 Mice*

[^18^F]SMBT-1 PET enables the detection of MAO-B, which is upregulated in reactive astrocytes in the human and rodent brain [31]. Here, we evaluated the distribution of [^18^F]SMBT-1 and its temporal and spatial relationships with amyloid-beta deposits in the brains of 5- and 10-month-old APP/PS1 mice and 11-month-old 3×Tg mice. First, we performed dynamic scans and evaluated the time-activity curve of [^18^F]SMBT-1 in the WT mouse brain. The cerebellum (gray line) showed faster washout and lower uptake at 45 min postinjection than did the other brain regions examined (SFig. 3). We compared cerebellar uptake in all the mouse groups and found no difference in the SUV (SFig. 4). Therefore, we chose 50–60 min after injection of [^18^F]SMBT-1 for acquiring the static scans in the following experiment and used the cerebellum as reference region for SUVR calculation.

**Fig. 3:**
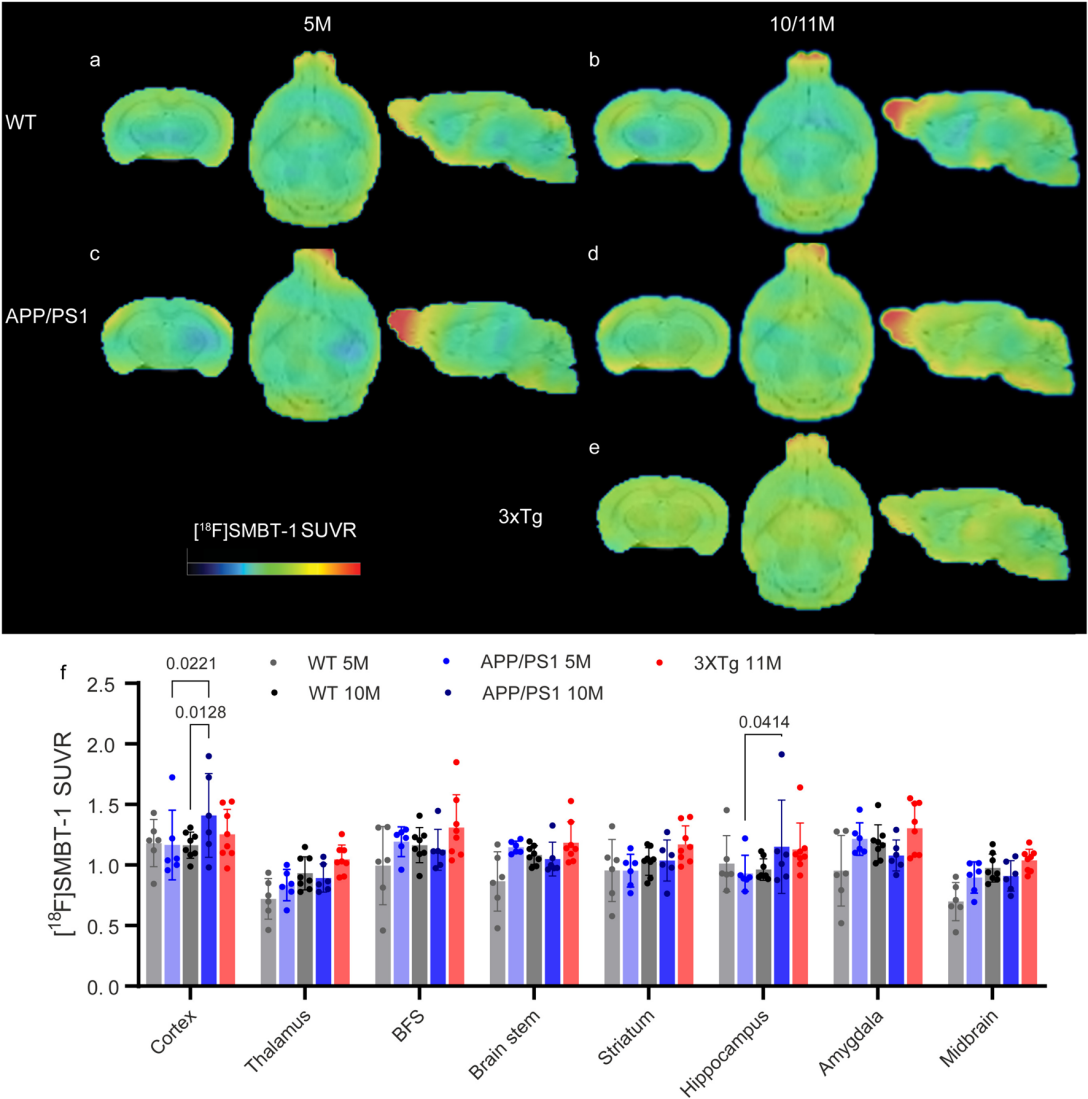
Increased [^18^F]SMBT-1 brain uptake in 10-month-old APP/PS1 mice compared to age-matched wild-type mice. **a-e** Images of SUVRs from 5- and 10-month-old wild-type (WT, **a**, **b**), 5- and 10-month-old APP/PS1 (**c**, **d**), and 11-month-old 3×Tg mice (**e**). The SUVR scale was 0–2.2. **f** Quantification of [^18^F]SMBT-1 in WT, APP/PS1 and 3×Tg mice using Cb as the reference region. BFS, basal forebrain system; Cb, cerebellum

We observed that the [^18^F]SMBT-1 SUVR (Cb as the reference region) was greater in the cortex and hippocampus of 10-month-old APP/PS1 mice than in 5-month-old APP/PS1 and age-matched wild-type mice. No regional difference in [^18^F]SMBT-1 SUVR was observed between 11-month-old 3×Tg mice and age-matched wild-type mice (Fig. 3a-f). This indicated that the increase in the level of astrocytic MAO-B accompanied amyloid accumulation in the brains of the APP/PS1 mice. [^18^F]SMBT-1 uptake (SUVR) in the olfactory bulb was rather high in all groups, with no significant between-group differences (SFig. 4c). No significant between-group differences was observed in [^18^F]SMBT-1 SUV in the cerebellum (SFig. 4 h).

### Glucose Metabolism Comparable Between APP/PS1 Mice and Wild-Type Mice

**Fig. 4:**
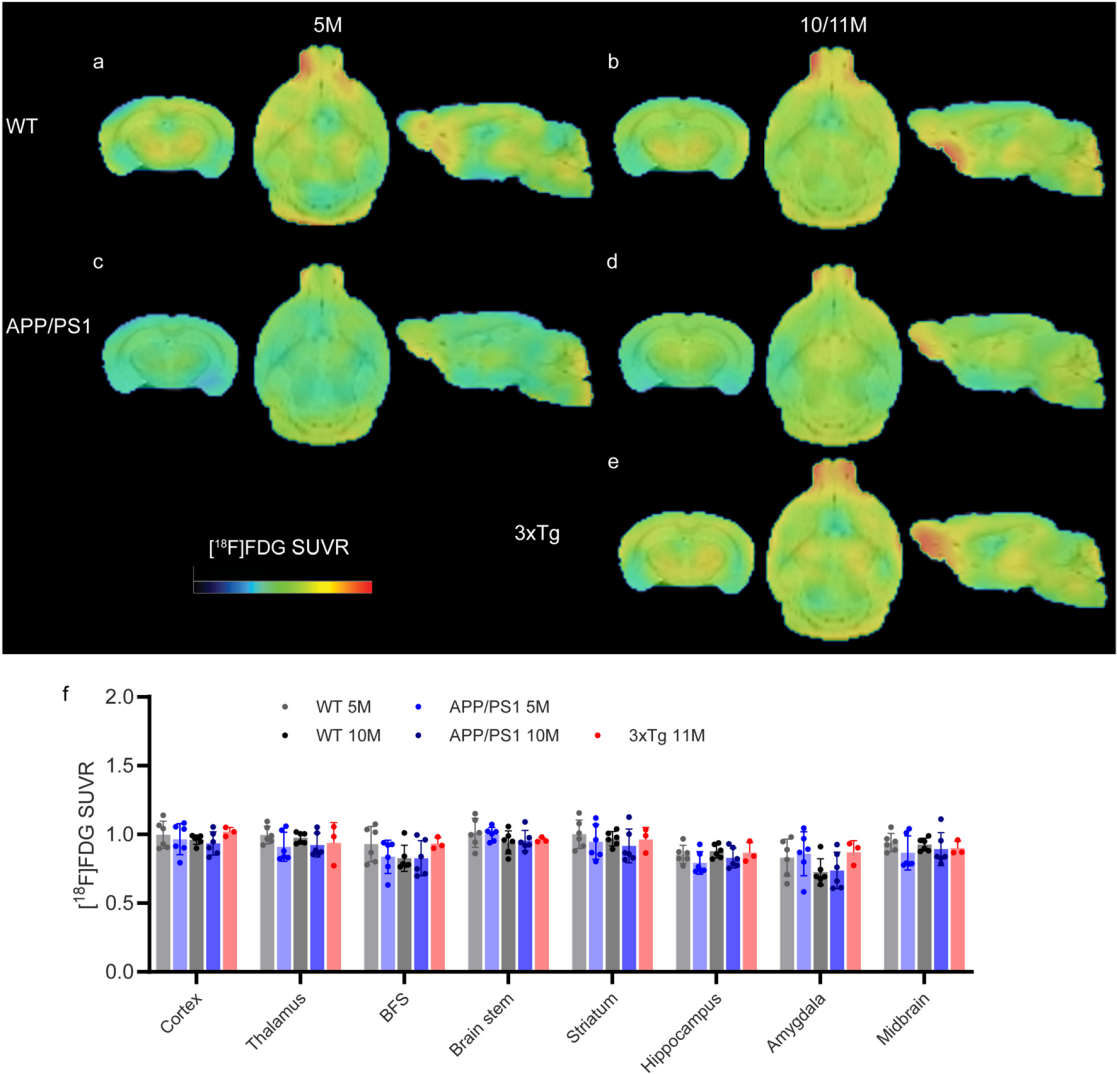
[^18^F]FDG brain uptake was lower in 5-month-old APP/PS1 mice than in age-matched wild-type mice. **a-e** Images of SUVRs from 5- and 10-month-old wild-type (WT, **a**, **b**), 5- and 10-month-old APP/PS1 (**c**, **d**), and 11-month-old 3×Tg mice (**e**). The SUVR scale was 0–1.8. **f** Quantification of [^18^F]FDG using Cb as the reference brain region in WT, APP/PS1, and 3×Tg mice. BFS, basal forebrain system; Cb, cerebellum

To assess the changes in cerebral glucose hypometabolism and if there are associations between these changes and other readouts, we performed [^18^F]FDG imaging in APP/PS1 mice, 3⨯Tg mice and WT mice at 5 and 10-11 months. The cerebellum was chosen as the reference region because it was used in earlier studies [74]. The [^18^F]FDG SUVRs (Cb as a reference region) were comparable in different brain regions between 5-month-old and 10-month-old APP/PS1 mice and age-matched wild-type mice (Fig. 4). No regional difference in [^18^F]FDG SUVR was observed in the brains of 11-month-old 3×Tg mice compared to age-matched wild-type mice (Fig. 4). No significant between-group differences was observed in [^18^F]FDG SUVR in the olfactory bulb (SFig. 4d). No significant between-group differences were observed in [^18^F]FDG SUV in the cerebellum (SFig. 4i).

### *[*^*18*^*F]DPA-714 SUVR Did Not Differ Among APP/PS1 Mice, 3*×*Tg Mice and Wild-Type Mice*

**Fig. 5:**
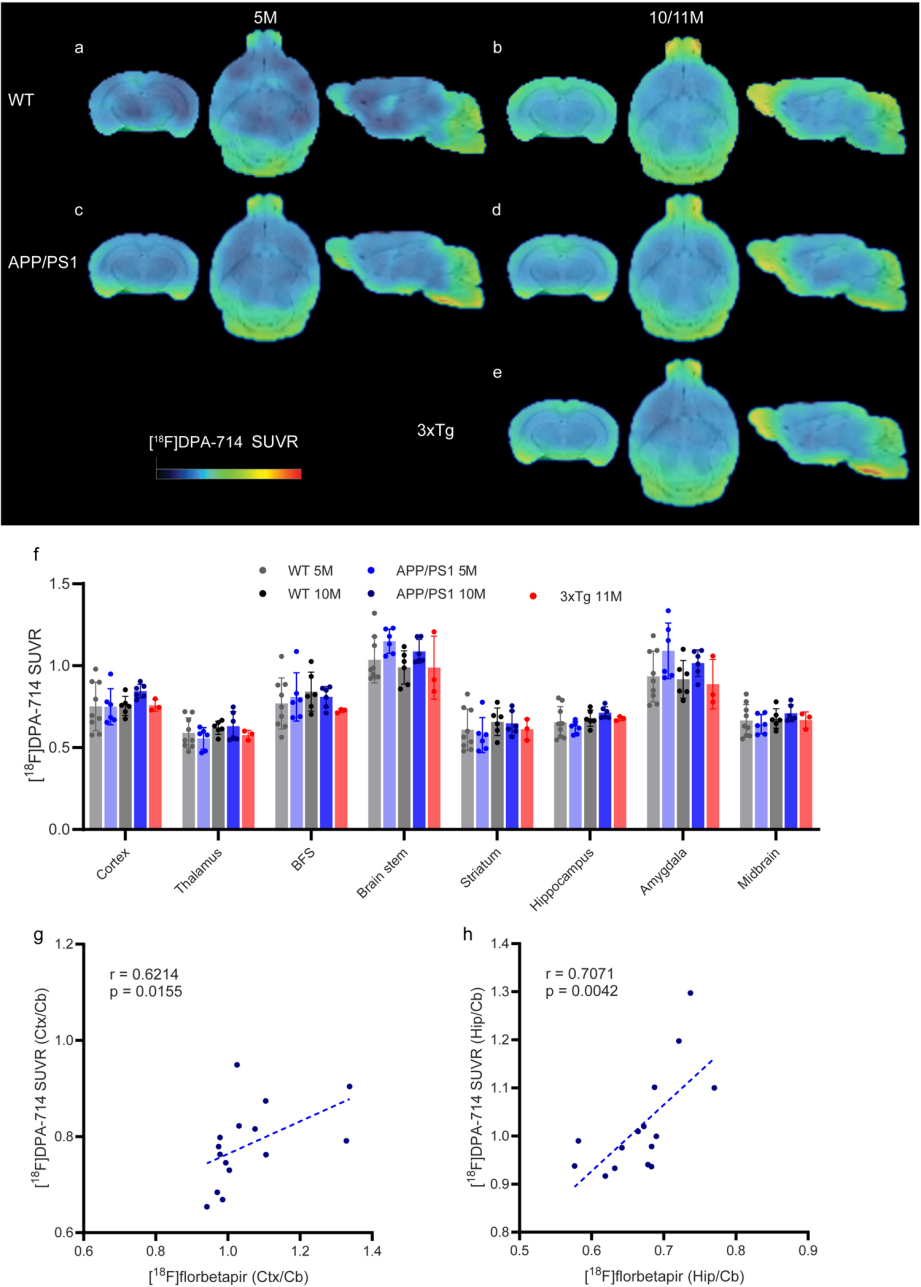
Comparable [^18^F]DPA-714 brain uptake in 5-month- and 10-month-old APP/PS1 mice, age-matched wild-type mice and 3×Tg mice. **a-e** Images of SUVRs from 5- and 10-month-old wild-type (WT, **a**, **b**), 5- and 10-month-old APP/PS1 (**c**, **d**), and 11-month-old 3×Tg mice (**e**). The SUVR scale was 0–2.2. **f** Quantification of the regional [^18^F]DPA-714 SUVR using Cb as the reference brain region in WT, APP/PS1 and 3×Tg mice. **g, h** Correlations between [^18^F]florbetapir SUVR and [^18^F]DPA-714 SUVR in the cortex (Ctx) and hippocampus (Hip) of mouse brains were assessed using Cb as the reference brain region. BFS, basal forebrain system; Cb, cerebellum

PET of TSPO tracers, such as [^18^F]DPA-714, has been widely used as an imaging biomarker for indicating microglial activation and neuroinflammation. Next, we assessed the pattern of TSPO by PET using [^18^F]DPA-714 in 5- or 10-month-old APP/PS1 mice and 11-month-old 3×Tg mice. Different reference brain regions, including the cerebellum [8, 38, 50], hypothalamus [41] and midbrain [22] (to avoid spillover), have been used for [^18^F]DPA-714 SUVR quantification in mouse models. Here, we used the Cb as the reference brain region for [^18^F]DPA-714 according to earlier studies using [^18^F]DPA-714 and [^11^C]PBR28 in mouse models [50], despite the known rather high signal in the cerebellum. No difference in [^18^F]DPA-714 SUVR was observed between 5- or 10-month-old APP/PS1 mice and age-matched wild-type mice (Fig. 5). No difference in [^18^F]DPA-714 SUVR was observed between 11-month-old 3×Tg mice and age-matched wild-type mice (Fig. 5). No significant between-group differences was observed in [^18^F]DPA-714 SUVR in the olfactory bulb (SFig. 4e). No significant between-group differences was observed in [^18^F]DPA-714 SUV in the cerebellum (SFig. 4j).

### *Association Between [*^*18*^*F]Florbetapir, [*^*18*^*F]PM-PBB3, [*^*18*^*F]SMBT-1, [*^*18*^*F]FDG, and [*.^*18*^*F]DPA-714*

To assess the spatial association between different pathologies, nonparametric Spearman’s rank correlation analysis was performed on the regional SUVR readouts for [^18^F]SMBT-1, [^18^F]florbetapir [^18^F]PM-PBB3, [^18^F]FDG, and [^18^F]DPA-714 within the transgenic group. Although the [^18^F]DPA-714 level was not significantly different between the transgenic and WT groups, positive correlations were observed between [^18^F]florbetpair SUVR and [^18^F]DPA-714 SUVR in the cortex (r = 0.6214, *p* = 0.0155, n = 15) and in the hippocampus (r = 0.7071, *p* = 0.0042, *n* = 15) among the transgenic mice (both APP/PS1 and 3×Tg mice; Fig. 5g, h). Positive correlations were observed between the [^18^F]florbetpair SUVR and [^18^F]DPA-714 SUVR in the cortex (r = 0.6014,* p* = 0.0428; *n* = 12) and in the hippocampus (r = 0. 7273, *p* = 0.0096, *n* = 12) within the APP/PS1 mice. No other regional correlation was found between different readouts.

### Reactive Astrocytes and Microgliosis with Tau Inclusions and Aβ Deposits

**Fig. 6:**
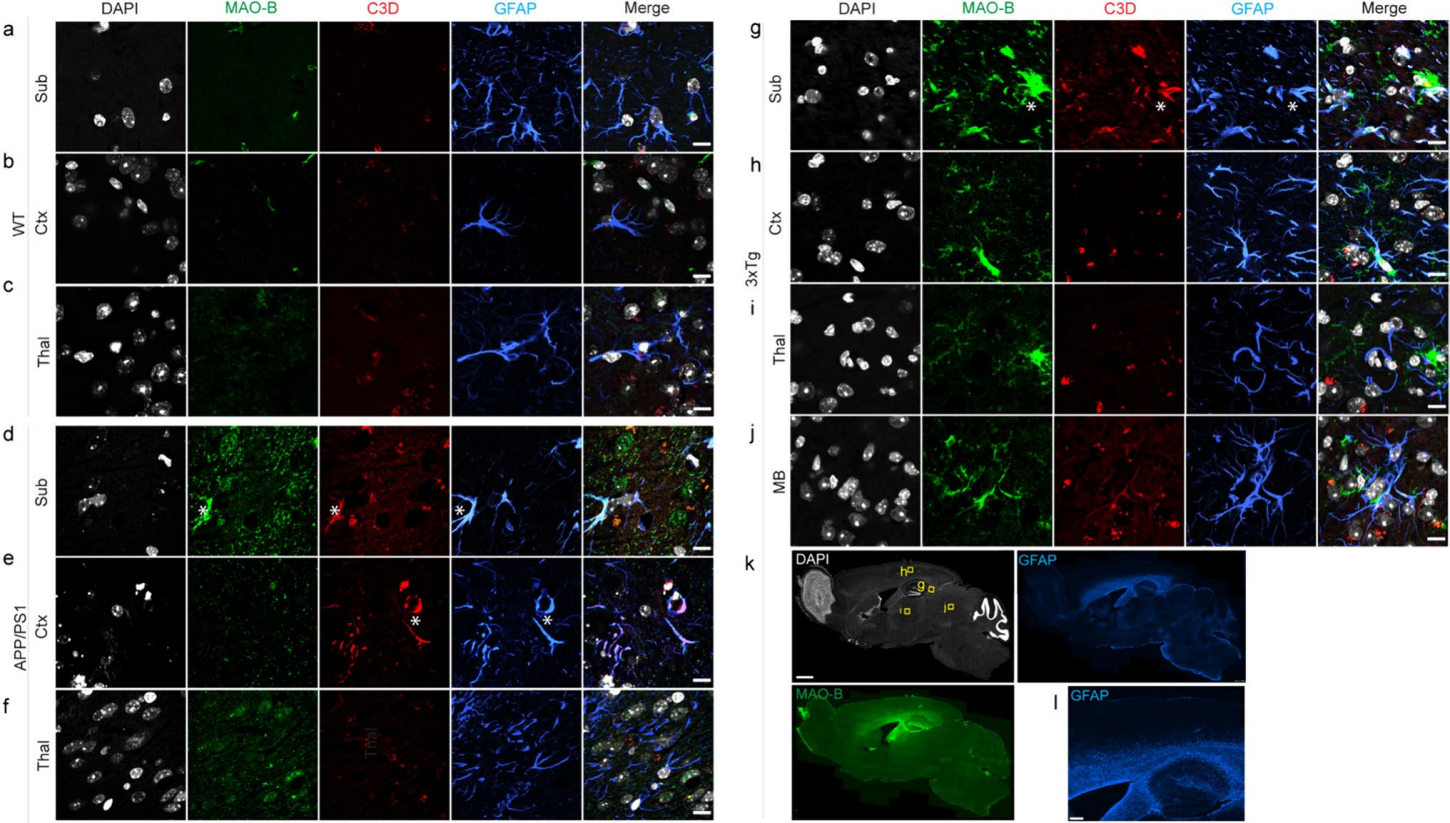
Immunofluorescence staining of MAO-B and astrocyte markers in mouse brains. Brain tissue sections from wild-type (WT), APP/PS1 and 3×Tg mice were stained for MAO-B (green)/C3D (red)/GFAP (blue). **a-j** Zoomed-in view showing the colocalization of MAO-B on C3D-positive astrocytes in the subiculum (Sub), cortex (Ctx), thalamus (Thal), and midbrain (MB). **k-l** Overview of the staining in 3×Tg mice showing the location of the regions (**g**, **h**, **i**, **j**). Nuclei were counterstained with DAPI (gray). * indicates colocalization. Scale bar = 10 μm (**a**–**j**)**,** 1 mm (**k**), and 200 μm (**l**)

Next, we evaluated the distributions of MAO-B and TSPO along with the astrocytic markers GFAP/C3d and Aβ deposits (6E10) and GluT1 in brain tissue slices. Given the age of the mice, autofluorescence of the brain tissue was assessed (SFig. 5). Only autofluorescence affects the DAPI channel due to the presence of amyloid-beta plaques (a representative image shows the subiculum of a 3×Tg mouse). MAO-B was detected on astrocytes via colocalization with C3D immunofluorescence in the cortex and hippocampus of APP/PS1 and 3×Tg mice and was found to colocalize with A1 reactive astrocytes (Fig. 6b, c). The MAO-B distribution appears to be greater in the white matter than in the gray matter of the mouse brain. The MAO-B signal in the cerebellum was low, indicating that the cerebellum is suitable as a reference brain region.

**Fig. 7:**
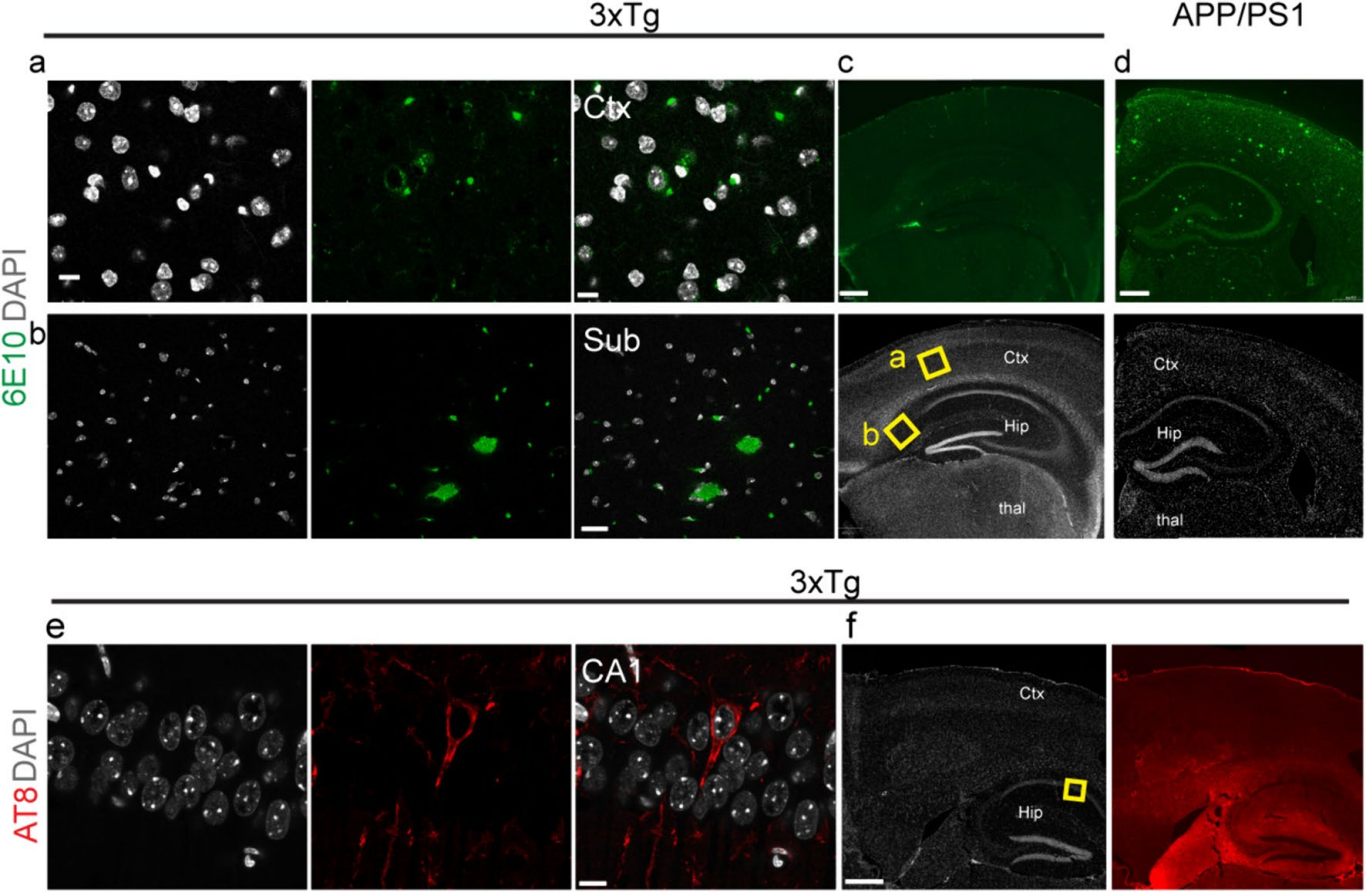
Limited amyloid-beta deposits and tau inclusions in the brains of 3×Tg mice and amyloid-beta plaques. **a-c** Limited amyloid deposits were observed in the subiculum and cortex (layer 3/4) brain tissue sections of 11-month-old 3×Tg mice stained for 6E10 (mainly intracellular); yellow squares in **c** indicate the locations of the zoomed-in view (**a**, **b**). **d** Amyloid deposits were abundant in the cortex (Ctx) and hippocampus (Hip) and in the thalamus (Thal) of 10-month-old APP/PS1 mice. **e**, **f** Limited tau inclusion was observed in the hippocampus (CA1) of 3×Tg mice stained for AT-8. The yellow squares in f indicate the locations enlarged in view (**c**). The anti-amyloid antibody 6E10 (green) and the anti-phospho-Tau antibody AT-8 (red) were used. Nuclei were counterstained with DAPI (white). Scale bars = 10 mm (**a**, **b**, **e**) and 400 mm (**c**, **d**, **f**)

In 3 × Tg mice at 11 months, limited Aβ deposits (mainly intracellular) and tau inclusions were observed, mainly in the subiculum and CA1 region of the hippocampus (Fig. 7a-c, e, f), validating the lack of amyloid PET ([^18^F]florbetapir, [^11^C]PIB) and tau PET ([^18^F]PM-PBB3) updates in the mouse brain. This finding is different from the abundant amyloid deposits in the cortex, hippocampus and thalamus of APP/PS1 mice at 10 months (Fig. 7d). Similarly, the levels of the glucose transport protein GluT1 were detected in the cortex and hippocampus of 3×Tg mice and wild-type mice (SFig. 6).

## Discussion

Here, we demonstrated increased brain regional [^18^F]SMBT-1 and [^18^F]florbetapir brain uptake in 10-month-old APP/PS1 mice and comparable [^18^F]FDG and [^18^F]DPA-714 uptake compared to age-matched wild-type mice. Moreover, [^18^F]DPA-714 uptake correlated with [^18^F florbetapir in the cortex and hippocampus, whereas no correlation was found between the uptake of [^18^F]SMBT-1 and other tracers in the brain.

For amyloid imaging, several microPET studies using [^18^F]florbetapir [7, 20, 64, 84], [^18^F]florbetaben, [^11^C]PIB [54, 55], and [^18^F]fluotemetamol in APP/PS1 mice have been reported. Our finding of increased [^18^F]florbetapir SUVRs in the cortex and hippocampus was in line with the known Aβ aggregate distribution and immunofluorescence staining in the brains of APP/PS1 mice. Our observation of a lack of increase in [^18^F]florbetapir uptake in 3**×**Tg mice is in line with previous observations using [^11^C]PIB (or [^18^F]florbetaben) [62] in 3**×**Tg mice at 4–16 months [15, 16] but differs from two other studies showing an increase at 8 and 10 months [15, 78]. We also found a limited distribution of Aβ (6E10) immunoreactivity in the brains of 11-month-old 3**×**Tg mice (few in the subiculum). Although high loads of amyloid and tau were observed in the original study [59], a recent study in 3**×**Tg mice from LaFerla lab showed that there is a lack of Thioflavin-S-positive amyloid staining in the brains of 12-month-old 3**×**Tg mice, likely due to genetic drift [35]. For tau imaging, PET has been performed using [^11^C]PBB3, [^18^F]PM-PBB3, and [^18^F]PI-2620 in PS19 and rTg4510 mice [4, 9, 22, 33, 42, 56, 61], as well as [^11^C]THK5317 [25] and [^11^C]THK5117 [12], which bind to both tau and MAO-B, in double mutant TgF344 rats. Thus far, only one ex vivo [^18^F]flortaucipir autoradiography study of aged APP/PS1 mouse brain slices with positive result [52]. We observed no change in the [^18^F]PM-PBB3 SUVR in the APP/PS1 mice at 5 months or in 3**×**Tg mice. Our AT-8 immunofluorescence staining showed that the tau inclusions in the brain of 3**×**Tg mice was rather limited. Many reports have shown that 3**×**Tg mice exhibit significant neurofibrillary tangles in the brain at this age [18, 59]; however, a recent study in 3**×**Tg mice from the LaFerla lab showed that there is a lack of phospho-Tau (AT-8) positive staining but high HT-7-positive total tau immunoreactivity in the hippocampus of 3**×**Tg mice at 12 months [35]. Therefore, the difference in the tau load might be due to genetic drift and the choice of antibody.

We found increased cortical and hippocampal MAO-B levels in 10-month-old APP/PS1 mice compared to wild-type mice but no difference in 11-month-old 3**×**Tg mice according to [^18^F]SMBT-1 PET. No earlier study has reported MAO-B imaging results in APP/PS1 mice. Nevertheless, an early increase in MAO-B has been reported in the thalamus of PS2APP mice at 5, 13, and 19 months and in the hippocampus at 14 and 19 months compared with that in wild-type mice by using [^18^F]F-DED [3], and in 6-month-old APPswe mice preceding amyloid plaque deposition using [^11^C]AZD2184 [66] using [^11^C]DED. Our lack of difference in [^18^F]SMBT-1 uptake in 3**×**Tg mice is in line with the findings of a recent study in which [^11^C]DED was used to evaluate the hippocampus or cortex of 10-month-old 3**×**Tg mice. However, another study using [^18^F]sulforhodamine-101 showed that uptake increased in 9- to 10-month-old 3**×**Tg mice [45]. Notably, [^18^F]Sulforhodamine-101 detects the thyroid hormone transporter OATP1C1, which is located mainly on astrocytes and endothelial cells, unlike [^18^F]SMBT-1, which targets MAO-B located on astrocytes and neurons.

Inconsistent results have been reported for [^18^F]FDG updates in animal models of AD, partly due to differences in the imaging protocol, fasting, anesthesia depth, sex, age, and heterogeneity between animals. Here, we found no difference in [18F]FDG uptake between 5- or 10-month-old APP/PS1 mice or between 11-month-old 3×Tg mice and wild-type mice. Higher [^18^F]FDG uptake in the brain of increase in 2, 3, 5, and 8 months old APP/PS1 mice [47] as well as 12-month-old APP/PS1 mice [65, 74] has been reported. While several other studies found that [^18^F]FDG uptake was lower in the brain of 6-month-old [68] and 11-month-old 3×Tg mice [1] than in WT mice; In addition, one study showed that there was no difference in [^18^F]FDG uptake detected in the brain of 12 months 3×Tg mice [58] compared to WT mice.

TSPO is overexpressed on activated macrophages and microglia and is considered a biomarker of neuroinflammation [46]. TSPO tracers [46, 87], including 1st generation [^11^C]PK11195, 2nd generation [^18^F]DPA-714, [^11^C]PBR28, and 3rd generation [^18^F]GE-180, have been the most widely used. Notably, the increase in the TSPO PET signal does not necessarily indicate microglial proliferation. [^18^F]DPA-714 showed favorable binding potential and selectivity and low nonspecific binding compared to [^11^C]PK11195 [14]. We observed no difference in the brain regional [^18^F]DPA-714 SUVR (Cb as reference region) between 5- or 10-month-old APP/PS1 mice and wild-type mice. However, a positive correlation was observed between [^18^F]DPA-714 and [^18^F]florbetapir SUVR in the cortex and between [^18^F]DPA-714 and hippomcampus in APP/PS1 mice and in APP/PS1 combined with 3×Tg mice. These findings indicate a close link between microgliosis and amyloid deposition. Several imaging studies have reported an increase in TSPO levels in APP/PS1 mice; [^11^C]PK11195 uptake increased at 16–19 months APP/PS1 mice (not at 13–16 months) compared to WT mice [79]; [^18^F]GE180 uptake increased at 26 months APP/PS1 mice compared to 4 months APP/PS1 mice [48]; [^18^F]DPA-714 uptake increased at 12 months [75] and at 18 months APP/PS1 mice compared to WT mice [11]. For 3**×**Tg mice, one study of [^11^C]PK11195 showed that the level was comparable to control at 4–16 months [16], while another study revealed an increase in the hippocampus at 10 months compared to wild-type mice using [^125^I]CLINDE [78].

This study has several limitations. First, the mice were cross-sectional, not longitudinal. Only 3×Tg mice of one age were chosen. The sample size of the 3**×**Tg mouse group and the sex balance of the animals were not optimal. Different 3×Tg mice were used for in vivo imaging and ex vivo staining. Moreover, we did not provide detailed information on the morphology or heterogeneity of astrocytes and microglia or whether the astrocytes were vessel associated or associated with pathology. Notably, there are distinct dynamic profiles of microglial activation [30] and reactive astrocytes between human and mouse models [19].

## Conclusion

Here, we showed increased levels of [^18^F]SMBT-1 and [^18^F]florbetapir in the brains of 10-month-old APP/PS1 mice compared to age-matched wild-type mice, preceding changes in the level of [^18^F]DPA-714. The [^18^F]florbetapir and [^18^F]DPA-714 SUVRs correlated in the hippocampus and cortex of the transgenic mice.

## Supplementary Information

Below is the link to the electronic supplementary material.

Supplementary file1 (DOCX 4139 KB)

## Data Availability

The datasets generated and/or analyzed during the current study are available from the corresponding author upon reasonable request.
