# Supplementary material for: Relationship Between Reactive Astrocytes, by [18F]SMBT-1 Imaging, with Amyloid-Beta, Tau, Glucose Metabolism, and TSPO in Mouse Models of Alzheimer’s Disease
Source: Mol Neurobiol. 2024 Mar 19;61(10):8387–401. doi: 10.1007/s12035-024-04106-7 (PMC11415417; doi:10.1007/s12035-024-04106-7)
Supplement: Supplementary file 1 — Supplementary file1 (DOCX 4139 KB) [file 12035_2024_4106_MOESM1_ESM.docx]

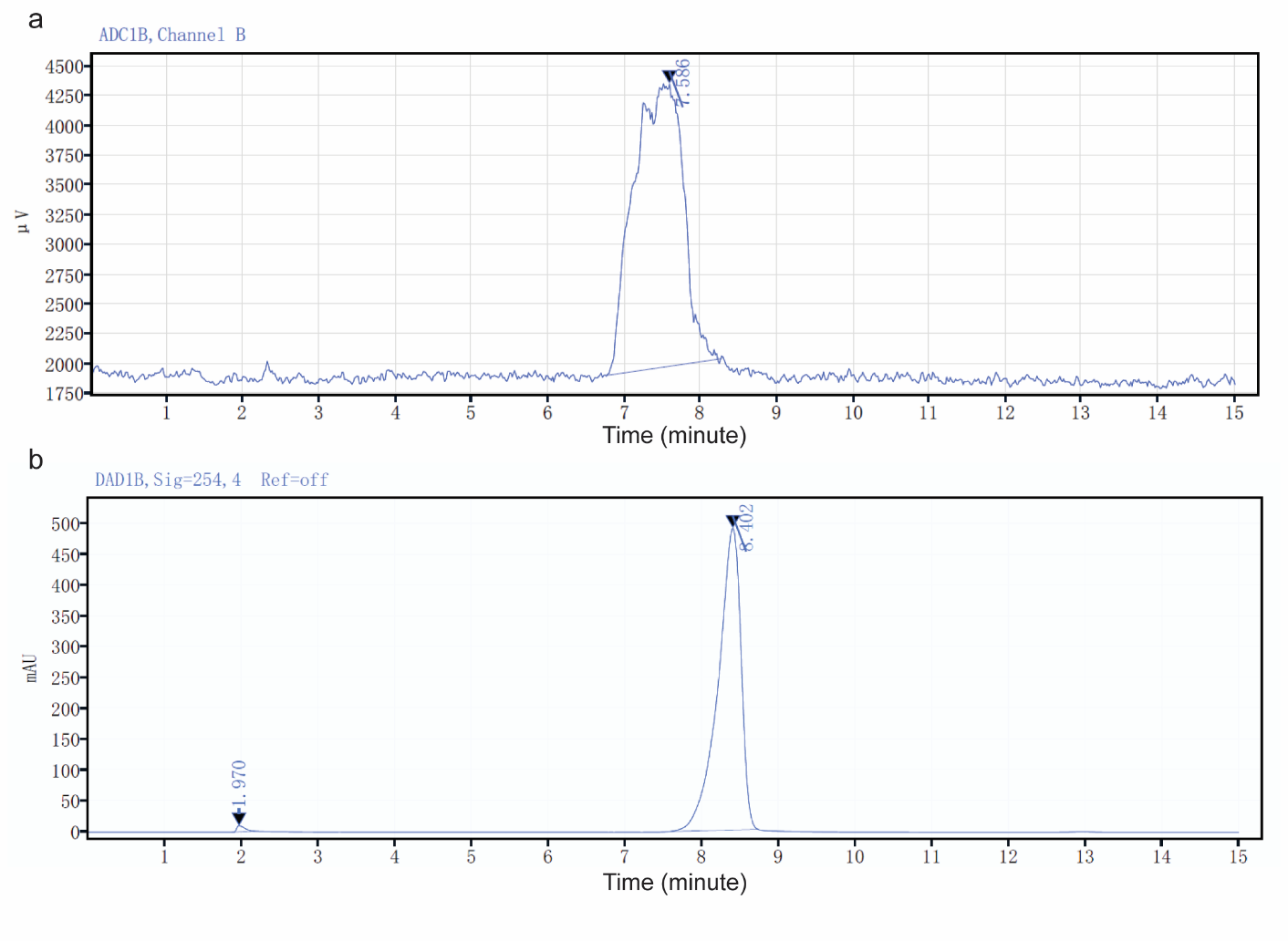


**Supplementary Fig 1. HPLC chromatogram of synthesized [^18^F]SMBT-1 and standard. (a)** Synthesized SMBT-1, (**b**) Standard.


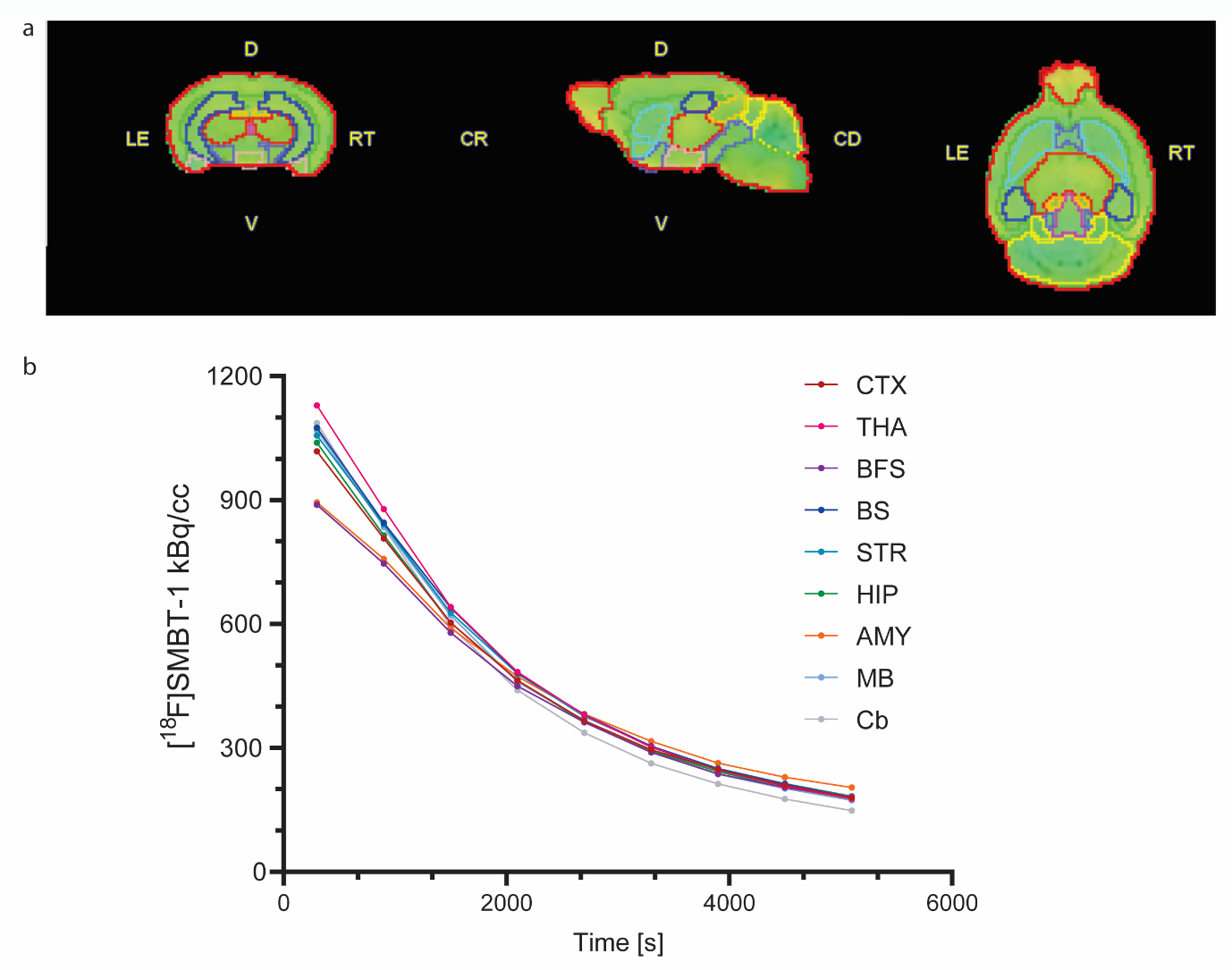
**Supplementary Fig 2. Volume-of-interest analysis and time activity curve of [^18^F]SMBT-1 in a mouse brain.** (**a**) Coronal, sagittal and horizontal views of a mouse brain. (**b**) Regional [^18^F]SMBT-1 time activity (kBq/cc) in the brain. CTX, cortex; THA, thalamus; BFS, basal forebrain system; BS, brain stem; STR, striatum; HIP, hippocampus; AMY, amygdala; MB, midbrain; Cb, cerebellum.


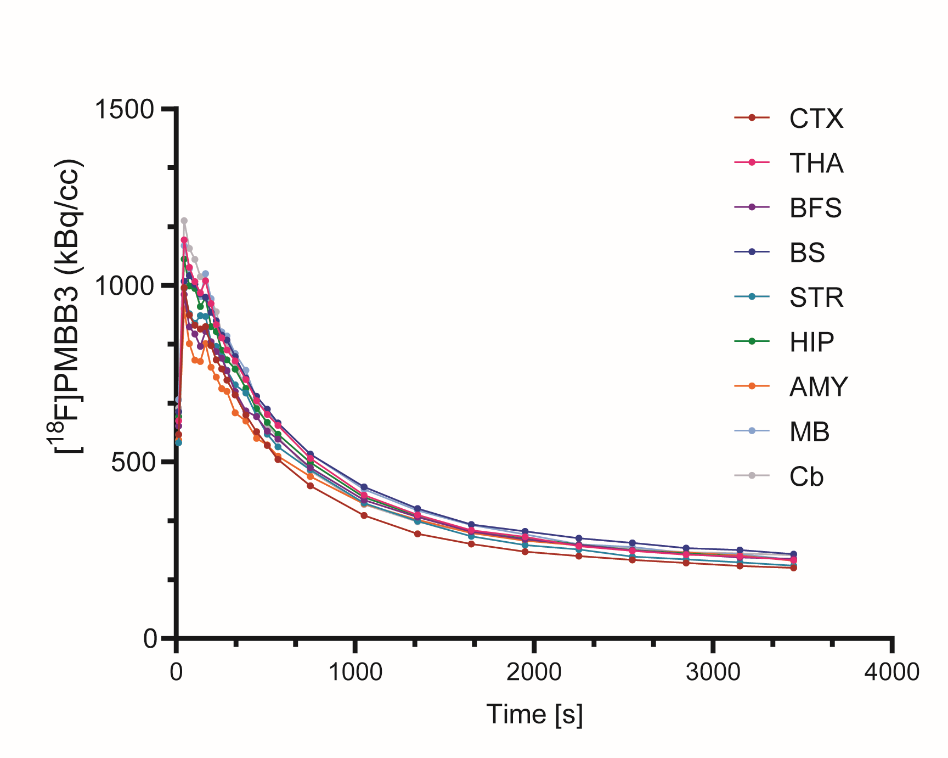


**Supplementary Fig 3. Time activity curve of [^18^F]PM-PBB3 in the mouse brain.** Regional time activity (kBq/cc) in the brain CTX, cortex; THA, thalamus; BFS, basal forebrain system; BS, brain stem; STR, striatum; HIP, hippocampus; AMY, amygdala; MB, midbrain; Cb, cerebellum.


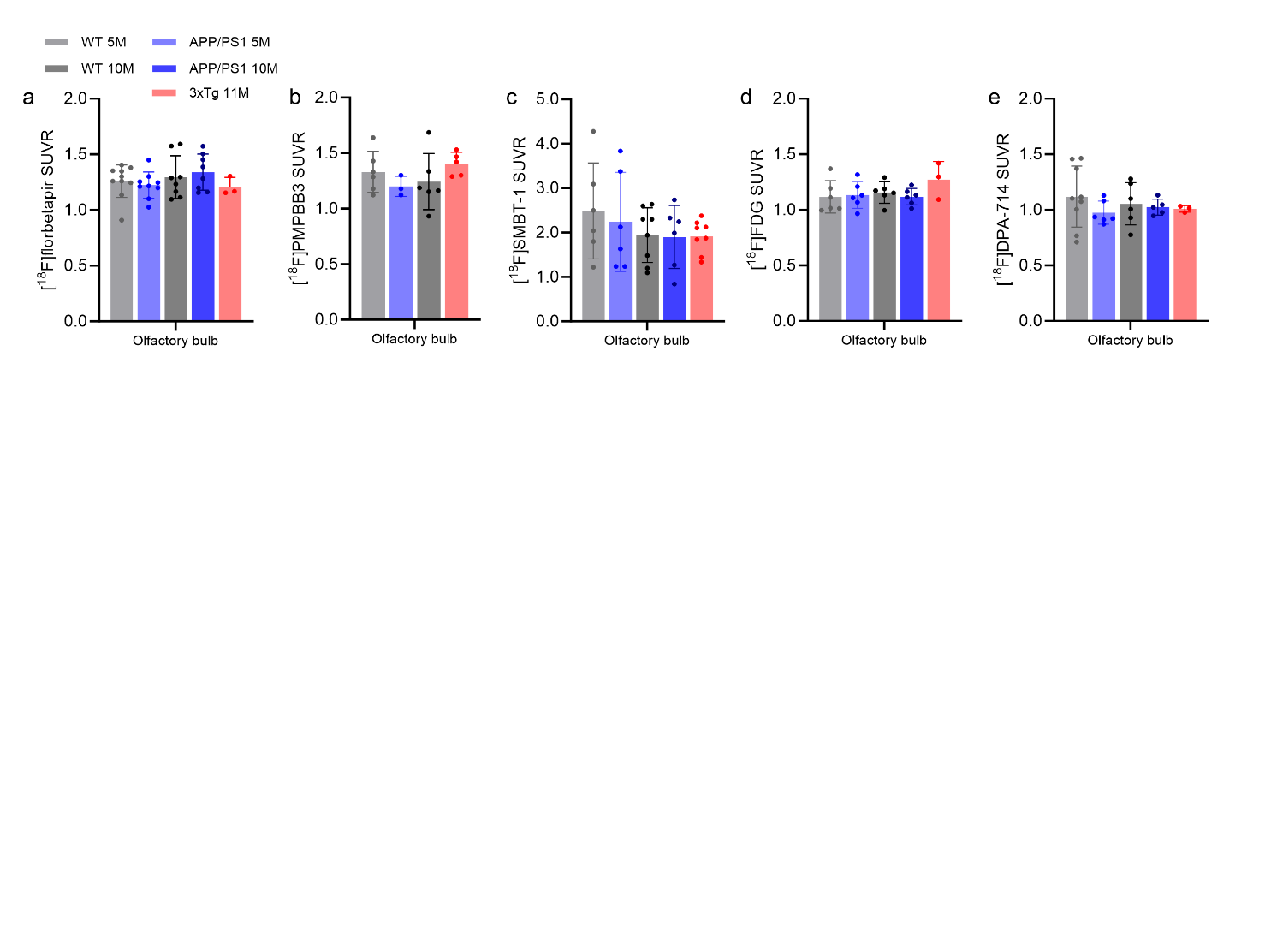


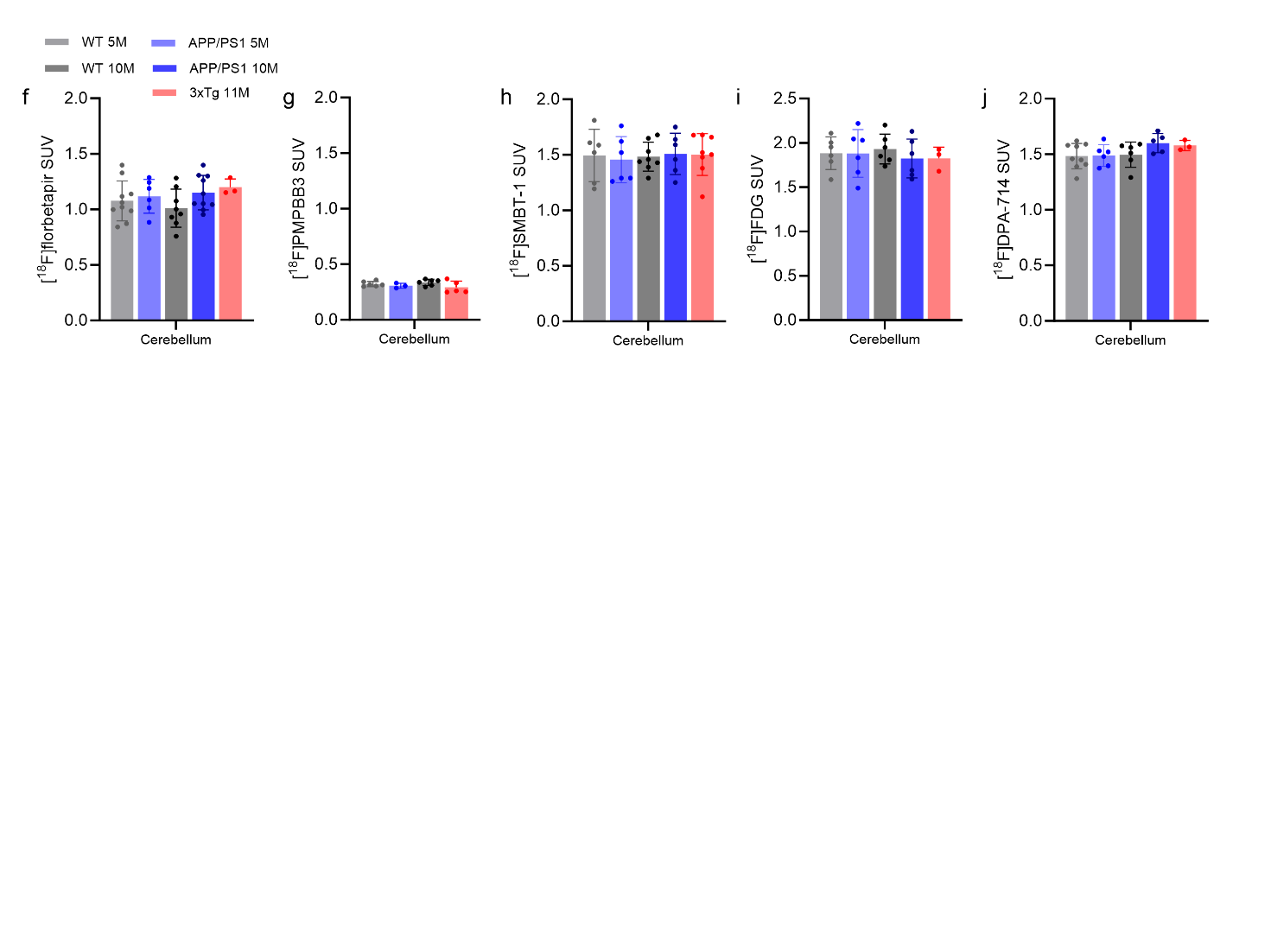
**Supplementary Fig 4. Summary of SUVR of different tracers in the olfactory bulb and SUV of different tracers in the cerebellum of mice.** (**a-e**) SUVR in the olfactory bulb with cerebellum as reference region; (**f-j**) SUV in the cerebellum of WT, APP/PS1 and 3×Tg mice. (a, f) [^18^F]florbetapir, (b, g) [^18^F]PM-PBB3, (c, h) [^18^F]SMBT-1, (d, i) [^18^F]FDG, (e, j) [^18^F]DPA-714.


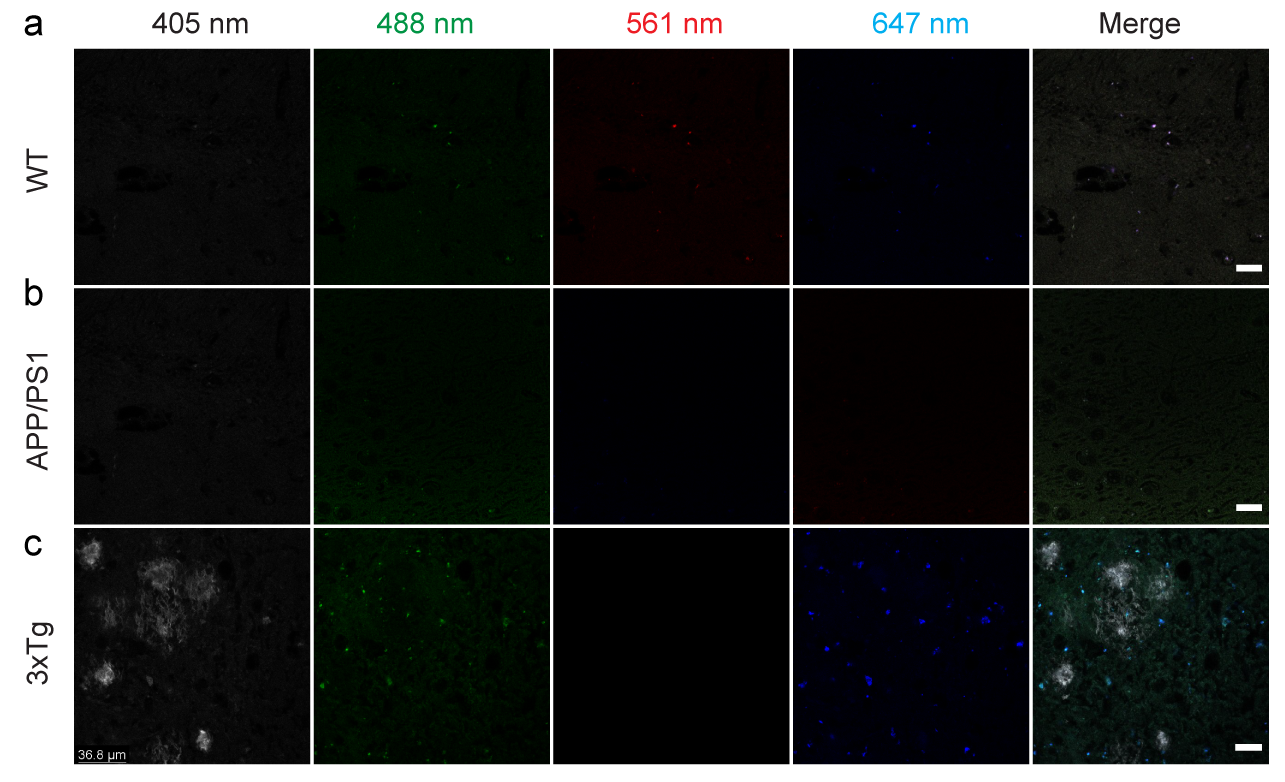


**Supplementary Fig. 5** **Examination of autofluorescence in the subiculum of difference mouse models**. (a-c) 405 nm, 488 nm, 561 nm, 647 nm and merged channels in the subiculum of WT, APP/PS1 and 3×Tg mouse brain. Signal was observed in the 405 nm (DAPI channel) on the subiculum of the 3×Tg mouse brain. The images were taken with the same confocal laser wavelength settings as that for the stained tissue sections shown in **Fig** 6. Scale bar = 20 μm.


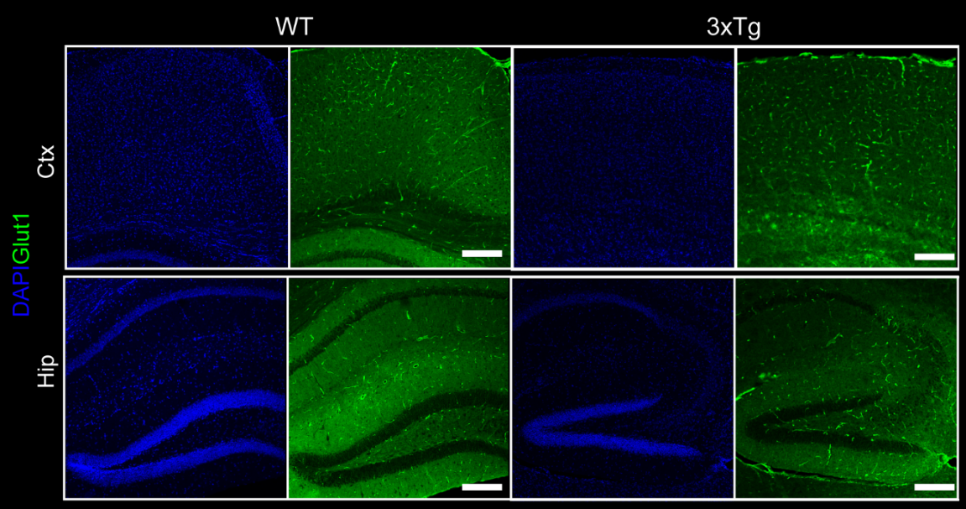


**Supplementary Fig 6. GluT1 staining in WT and 3×Tg mouse brains.** (**d**) Brain tissue sections of wild-type and 3×Tg mice were stained for GluT1 (green) in the cortex and hippocampus. Nuclei were counterstained with DAPI (blue). Scale bar = 200 μm.

**Supplementary Table 1 List of antibodies**

| **Antibody** | **Catalog no.** | **Dilution** | **Supplier** |
| --- | --- | --- | --- |
| Alexa fluor488 donkey anti-mouse IgG (H+L) | 715-545-151 | 1:500/1:250/1:125 | Jackson |
| Goat anti-mouse IgG (H+L) highly cross-adsorbed secondary antibody, Alexa fluor plus 488 | A32723 | 1:500 | Invitrogen |
| Cy3 donkey anti-rabbit IgG (H+L) | 711-165-152 | 1:250 | Jackson |
| Donkey anti-goat IgG (H+L) cross-adsorbed secondary antibody, Alexa fluor 546 | A11056 | 1:200/1:50 | Invitrogen |
| Alexa fluor647 donkey anti-guinea pig IgG (H+L) | 706-605-148 | 1:250 | Jackson |
| Donkey anti-rabbit IgG (H+L) highly cross-adsorbed secondary antibody, Alexa Fluor Plus 647 | A32795 | 1:200 | Invitrogen |
| DAPI (4',6-Diamidino-2-Phenylindole, Dihydrochloride) | D1306 | 1:1000 | Invitrogen |
| Mouse phosphor-Tau (Ser202, Thr205) monoclonal antibody (AT8) | MN1020 | 1:1000 | Invitrogen |
| Mouse Purified anti-β-Amyloid, 1-16 monoclonal antibody (6E10) | 803001 | 1:1000 | Biolegend |
| Goat complement component C3d polyclonal antibody | AF2655 | 1:200 | R&D Systems |
| Guinea pig GFAP polyclonal antibody | BP5082 | 1:1000 | OriGene |
| Mouse Glut1 (C-terminus) monoclonal antibody | MABS132 | 1:1000 | Milipore |
| Mouse MAO-B (D-6) monoclonal antibody | sc-515354 | 1:500 | Santa Cruz |
